# Discordant FeLV p27 immunoassay and PCR test results in 21 cats with hematologic disorders

**DOI:** 10.1177/1098612X231183297

**Published:** 2023-07-13

**Authors:** Matthew Kornya, Dorothee Bienzle, Janet Beeler-Marfisi

**Affiliations:** 1 Department of Clinical Studies, University of Guelph, Guelph, ON, Canada; 2 Department of Pathobiology, Ontario Veterinary College, University of Guelph, Guelph, ON, Canada

**Keywords:** Hematology, retroviruses, discordancy, laboratory medicine

## Abstract

**Case series summary:**

A total of 1692 medical records from a primary care feline practice and a veterinary referral hospital were evaluated retrospectively to assess discordant feline leukemia virus (FeLV) test results. In total, 73 cats were positive for FeLV using serum in a lateral flow immunoassay (LFI) or laboratory-based ELISA. Of these cats, 21 subsequently tested negative for FeLV proviral DNA by non-quantitative PCR on EDTA whole blood (16/21, 76.2%), bone marrow (4/21, 19%) or both (1/21, 4.7%). The proportional morbidity (an estimate of prevalence in a sample of the total population) for FeLV by LFI/ELISA and PCR assays was 3.1%, consistent with that reported in previous studies for cats in North America. Cats with discordant LFI/ELISA and PCR results had either primary bone marrow disease (18 autoimmune, one neoplastic), a bone marrow insult (hemotrophic mycoplasmosis) or systemic inflammation (pyothorax with a marked neutrophilic leukocytosis). The percentage of cats with a positive LFA/ELISA result and negative PCR assay surviving to discharge was 85.7% (18/21). Of these, 88.9% (16/18) survived 4 months to 6 years. Seven cats (33.3%) were re-tested with LFI or ELISA once primary disease was controlled, and all tested negative.

**Relevance and novel information:**

These findings indicate that in cats with bone marrow disease that shares features of progressive FeLV infection, positive LFI and ELISA FeLV test results should be followed up with FeLV proviral DNA PCR testing, particularly in populations where disease prevalence is low.

## Introduction

Feline leukemia virus (FeLV) is a ubiquitous retrovirus that infects cats worldwide.^1^ Transmitted through casual contact between cats,^2,3^ infection causes a wide range of clinical signs dependent on viral strain and the animal’s immune response.^1,4^ Diseases associated with FeLV include hematopoietic neoplasia, immune-mediated and degenerative diseases, and immunosuppression leading to secondary infection.^5^ The median survival time for cats with progressive infection is reported as 2–3 years from the time of diagnosis.^6,7^ Thus, given the seriousness of the effects of FeLV infection, test accuracy and correct interpretation are critical.

There are several diagnostic tests for FeLV, including lateral flow immunoassay (LFI) or ELISA for the p27 antigen, PCR for proviral DNA, immunofluorescent antibody testing and, less commonly, reverse transcription PCR for viral RNA, virus isolation or antibody titer.^3,8–11^ In-house LFI tests for FeLV p27 antigen are widely used and have a reported sensitivity of 63–100% and specificity of 94–100%.^8,12–14^ The reference laboratory ELISA (IDEXX Laboratories) may also include an additional step whereby positive samples are retested using a confirmatory assay to demonstrate p27-specific neutralization.^15^ Testing for FeLV proviral DNA by PCR is performed at reference laboratories, and although no true gold standard exists for the diagnosis of FeLV, it is an important follow-up test to determine a cat’s infection status.^1,12,16,17^

Clinical disease owing to FeLV is classically associated with ‘progressive infection’ characterized by high viral loads, a low antibody response and often a guarded long-term prognosis.^18^ Other manifestations of exposure can occur, including regressive infection, wherein a moderate immune response results in viral integration with the host genome but has minimal viral replication and prolonged survival without clinical signs; and focal infection wherein the disease is confined to a single organ.^1^ Animals with regressive or focal infection may develop clinical signs or shed virus in some situations where the host immune response is impaired.^1^ Since cats with regressive or focal infections have no obvious clinical signs, it has been thought that FeLV infection does not have a negative impact on their health. However, it is possible they may experience some degree of bone marrow suppression. A major question in FeLV diagnostic testing, therefore, is not simply ‘is the animal infected with FeLV’ but ‘is FeLV the cause of the clinical signs.’

Formerly a common cause of hematologic disease in cats,^19,20^ widespread testing, vaccination and segregation of positive cats has dramatically decreased the prevalence of FeLV infection in some parts of North America and Europe.^21–25^ Concomitantly, many cats with hematologic disease now have FeLV-negative test results on LFI or ELISA assays.^26–29^ While these facts suggest a decrease in the prevalence of progressive infection, regressive disease is more difficult to diagnose and may still remain highly prevalent.^30,31^ Patterns of positivity and degree of viral load may also vary and have an influence on survival and our ability to detect infection.^32^ However, retroviral status should be determined in all sick cats, and recommendations of the American Association of Feline Practitioners (AAFP) are to follow up positive FeLV LFI test results with an ELISA, PCR or IFA when needed.1 This is particularly important because positive FeLV results may be encountered in cats with bone marrow disease, which will typically be interpreted to indicate progressive FeLV infection. However, particularly in areas where disease prevalence is low, the possibility of false-positive test results is increased, and disease may be seen in animals with focal or regressive FeLV infection, which may be coincidental to their retroviral status. The brand of patient side test may also have an impact on false-positive results.^13^ Since many cats are euthanized based on a positive FeLV LFI/ELISA test result,^7^ differentiating true- and false-positive results is crucial. FeLV-positive cats may have a reasonable long-term prognosis and can often be successfully rehomed.^33^ It is also important that a veterinarian be able to differentiate a cat that is progressively infected and has clinical signs caused by FeLV from one that is regressively infected but has an unrelated concurrent condition, since prognosis and therapy may differ.

This case series describes 21 cats with hematologic disease and a positive FeLV serum p27 LFI/ELISA but negative PCR test result and, in a subset of cases, a negative subsequent LFI/ELISA test result.

## Case series description

Medical records between 2016 and 2022 from a primary care, feline-only hospital and a veterinary referral hospital were reviewed for cats that tested positive for FeLV in blood samples using an LFI device (SNAP FeLV/FIV; IDEXX) or a reference laboratory-based FeLV ELISA (IDEXX), but had negative results for FeLV proviral DNA with a non-quantitative assay (FeLV RealPCR; IDEXX) on either EDTA whole blood or bone marrow aspirates. Of the 21 PCR tests, 17 (81%) were performed on the same blood sample as the ELISA; in the four remaining cases, PCR testing was performed on samples collected 1–3 days later (median 1 day).

The signalment, presenting complaint, result of FeLV diagnostic testing, final diagnosis, treatment and outcome for each animal was retrieved from the records and entered into a database (Microsoft Excel 365). No cats had an FeLV PCR test performed in absence of a previous positive FeLV LFI/ELISA.

Over the study period, 1692 cats (1518 at the primary care hospital and 174 at the referral hospital) were tested for FeLV by LFI/ELISA. There were 45 and 28 positive test results from the primary care and referral hospital, respectively, for a total of 73/1692 (4.3%) cases. Of the LFI/ELISA FeLV-positive cats, 41 were male and 32 were female. The median age across the hospitals was 3.1 years (range 0.8–11.0). No statistically significant differences in patient signalment data were identified between hospitals. Two FeLV test-positive cats also tested positive for feline immunodeficiency virus (FIV) with the LFI. Seven cats (Table 1) had follow-up testing performed a median of 2 months (range 1–6) after initial testing.

**Table 1:** Care facility, test results, clinical diagnosis and outcome of cats with discordant FeLV test results

Case	Location*	Results	Diagnosis	Outcome
1	A	ELISA +, BMPCR–	IMHA	Unknown
2	A	ELISA +, BMPCR–	NRIMHA	Recovery
3	A	ELISA +, PCR–	IMHA	Recovery
4	A	ELISA +, PCR–, later LFI–	NRIMHA	Recovery
5	A	ELISA +, PCR–	Immune-mediated bicytopenia	Unknown
6	A	ELISA +, PCR–, IFA–	Immune-mediated bicytopenia	Recovery
7	B	LFI +, PCR–, later LFI–	*Mycoplasma hemofelis* infection	Recovery
8	B	LFI +, PCR–, later LFI–	NRIMHA	Recovery
9	B	LFI +, PCR–, later LFI–	Immune-mediated bicytopenia	Recovery
10	B	LFI +, BMPCR–	NRIMHA	Euthanasia
11	B	LFI +, PCR–	NRIMHA	Euthanasia
12	B	LFI +, PCR–	NRIMHA	Recovery
13	B	LFI +, PCR–, later LFI–	Pyothorax	Recovery
14	B	LFI +, PCR–	ALL with IMHA	Euthanasia
15	A	ELISA +, PCR–	NRIMHA	Recovery
16	B	LFI +, PCR–, later LFI–	Immune-mediated pancytopenia	Recovery
17	A	ELISA +, PCR–, BMPCR–	NRIMHA, myelofibrosis	Recovery
18	A	ELISA +, PCR–	Immune-mediated bicytopenia	Recovery
19	A	ELISA +, BMPCR–	Immune-mediated bicytopenia	Recovery
20	A	ELISA +, PCR–, later ELISA–	IMHA	Recovery
21	A	ELISA +, PCR–	NRIMHA	Recovery

* A = primary care feline practice; B = referral hospital

ALL = acute lymphocytic leukemia; BMPCR = bone marrow PCR; FeLV = feline leukemia virus; IMHA = immune-mediated hemolytic anemia; LFI = lateral flow immunoassay; NRIMHA = non-regenerative immune-mediated hemolytic anemia

All LFI tests were performed by clinicians or technicians experienced in laboratory methods according to the manufacturer’s instructions and standard operating procedures. Tests were visually interpreted without the use of an automated reader. In cases of very faint positive results, the test was repeated on the same sample and only twice positive results were considered positive.

Of the 45 primary care and 28 referral hospital cats with positive FeLV LFI or ELISA results, nine (20%) and 12 (43%) tested negative by FeLV PCR, respectively. In total, 21/73 (28.8%) LFI or ELISA FeLV-positive cats had negative proviral PCR test results. Results of the PCR assay agreed with the immunoassay in 52/73 cases, yielding a positive predictive value for detecting FeLV proviral DNA in a sample following an FeLV positive antigen result of 71.2%. The proportional morbidity (an estimate of disease prevalence in a sample of the total population) of LFI/ELISA and PCR FeLV-positive cats was 31/1462 (2.1%) in the primary care population and 21/230 (9.1%) in the referral population. Among cats with discordant FeLV LFI/ELISA and PCR test results, 13 were castrated males and eight were spayed females. The median age was 2.6 years (range 0.8–11.0).

Of the 21 cats with discordant test results, 14 were diagnosed by LFI, while the remainder were diagnosed by an ELISA at a reference laboratory (IDEXX). Test samples for proviral PCR were EDTA blood (n = 16), bone marrow (n = 4) and both blood and bone marrow (n = 1). In one cat with a positive FeLV LFI result, a subsequent bone marrow immunofluorescent antibody test (IDEXX) was negative. Initial LFI/ELISA testing was performed before or within the first 24 h of therapy in all cats. Bone marrow samples were collected within 3 days of diagnosis in animals with poorly controlled disease despite therapy. Results of immunoassay tests after disease recovery in seven initially immunoassay-positive cats were negative (Table 1). These included six initially LFI-positive results and one initially ELISA-positive result. No cats tested positive on repeat p27 testing.

Of 21 cats with discordant FeLV test results, 18 were diagnosed with immune-mediated hematologic disease (12 with anemia, five with bicytopenia and one with pancytopenia), one cat with acute lymphocytic leukemia (ALL) and two with infectious disease (hemotrophic mycoplasma and pyothorax with a marked neutrophilic leukocytosis). Of these 21 cats, three (14.3%) were euthanized during initial hospitalization, two for financial reasons and the cat with ALL because of a poor prognosis. In total, 18 (85.7%) cats were discharged from the hospital, of which 16 (88.9%) have survived between 4 months (the time of writing) to 6 years, and two (9.5%) cats were lost to follow-up. Four of these 16 cats have been weaned off all medications, including the two with infectious disease, but 12 (75%) remain on some form of immunosuppressive therapy. The disease condition, testing performed, type of hospital and outcome are listed in Table 1.

## Discussion

Historically, FeLV infection was considered the most common cause of bone marrow disease in cats. Indeed, most cats with acute leukemia and myelodysplastic syndromes were presumed to be FeLV-positive.^20,29^ While the rate of FeLV-positivity in cats with lymphoma has decreased substantially in recent years, it remains a primary differential diagnosis for cats with blood dyscrasia.^34,35^ It is possible that more cats are presenting with regressive, focal or other atypical manifestations of FeLV infection. It has also become evident that survival in cats is negatively correlated with viral load, and that cats with progressive infection tend to have higher proviral copy number and antigen concentration.^32^

In two cats in this series, concurrent FeLV and FIV infection may have affected the outcome. One of the FIV-positive cats had pyothorax and the other had non-regenerative anemia. In both cases, the FIV status could have affected the disease syndrome by predisposing to infection or autoimmune disease. No follow-up FIV testing was performed on these cats. However, as both cases recovered from their respective condition, the ‘dual positivity’ did not appear to negatively affect outcome, contrary to previous reports of a worse outcome in this population of cats.^36,37^

In four of the cats with FeLV, follow-up proviral PCR testing was performed on bone marrow and not blood. It is possible that an animal with focal, non-marrow infection (eg, localized to the spleen) may be blood PCR-positive and bone marrow PCR-negative, which might account for the discordance. However, since bone marrow aspirates contain abundant blood, marrow samples should also reflect FeLV provirus in peripheral blood.

All cats with discordant FeLV LFI/ELISA and PCR test results identified in two veterinary hospitals over a 6-year period had some form of marrow insult, disease or activation. Reasons for the discordant FeLV test results are unresolved from the available data but are similar to a previous report in two cats with immune hemolytic anemia.^38^ It is also possible that the cats in this series were truly positive, but with a regressive or focal form of infection. In this case, neither test would be erroneous, rather illustrative of the difficulty in classifying these patients based on limited testing.

Early in the course of infection, regressive FeLV infection may present with antigen positivity and a negative proviral PCR test result, although PCR on bone marrow would often be expected to be positive.^1^ Once the virus is integrated into the host genome, proviral PCR results would be expected to be positive. It is also possible for antigen positivity to wax and wane, and so follow-up LFI/ELISA may have been negative as a result. Therefore, the findings in this series might be attributed to regressive infection. While regressive infection has traditionally been thought not to be associated with clinical disease, in one publication it was suggested that bone marrow suppression may occur.^39^ However, that study suggested that bone marrow suppression in regressive infection was rare, and although associated with a p27 negative and proviral PCR positive status, causality was not determined.

Focal infection may be another explanation for discordant results. In this situation, the cat may be FeLV infected; however, the infection may be confined to a single organ and not involve the blood or bone marrow.^1^ Focal infection of the bone marrow may also occur; however, in this situation, the clinicopathologic findings would be reversed, with positive bone marrow PCR and negative p27 testing.^40^ In one report of cats with progressive FeLV infection, a higher FeLV RNA copy number was detected by real-time PCR of splenic samples than blood or bone marrow; however, it is not clear if this also applies to proviral DNA.^41^ Focal infection could result in antigen positivity with negative blood and bone marrow proviral PCR results. Confirmation of this happening would be difficult as it would require PCR testing of multiple organs in each cat.^42^ It is, however, difficult to conceptualize how focal infection could lead to the hematologic syndromes identified in this case series.

It is also possible that cats in this study were neither focally nor regressively infected, and that one of the tests was truly incorrect. In the light of the long-term wellbeing of the majority of cats with discordant results (Table 1), and the high sensitivity of the proviral PCR assay,^12^ it is possible that the LFI/ELISA result was erroneous and that cats did not have transiently integrated exogenous FeLV and antigenemia. In addition, if focal or regressive infection was present, it seems unlikely based on our current understanding of FeLV pathogenicity that it was the cause of disease in this population. The patient-side LFI has been reported to have a sensitivity and specificity of 100% at an FeLV prevalence of 1%, 5% and 10% relative to concordant positive results with two different laboratory-based ELISAs.^13^ In turn, one of the laboratory-based ELISAs (IDEXX) was reported to have 100% concordance with a quantitative PCR assay for exogenous FeLV and an alternative ELISA.^15^ However, in relation to a different quantitative PCR assay, sensitivity and specificity of the LFI were reported as 63 and 94%, respectively.^28^ However, these studies were likely inadequate for detecting regressive infection, as there was only a single antigen test performed at one time point. Furthermore, it appears unlikely that an inappropriate cycle threshold cutoff in the quantitative PCR assay, or lack of binding of primers to exogenous FeLV sequences, would generate a false-negative PCR result.In general, FeLV p27 antigen concentrations have been shown to correlate with proviral loads by PCR.^43^ Rather, an LFI/ELISA-interfering substance in the blood of cats with profound inflammation or immune activation is a possibility that should be explored. The original monoclonal antibodies against FeLV p27, still in current use, were highly specific for feline rather than other species’ retroviruses.^44^ Hemoglobin, bilirubin or lipid added to test samples, and prior immunization of cats with mouse IgG, was also reported to not affect performance of the FeLV ELISA.^15^ An alternate hypothesis whereby both the ELISA and PCR were true-positive and -negative results, respectively, might be conceivable if marked immune activation or inflammation could have eliminated all or most FeLV-infected hemolymphatic cells. Serial immunoassays and PCR tests would be required to distinguish between erroneous test results and the latter hypothesis.

Positive predictive value (PPV) and negative predictive value of binary tests are highly dependent on prevalence, which was undetermined in this population of cats with discordant test results. Seropositivity among North American cats overall is approximately 3%, which is similar to the proportional morbidity identified in the general practice population here.^21^ Using this value for ‘true’ prevalence, and sensitivity and specificity of 96.6% and 98%, respectively,^45^ the PPV for the FeLV LFI (IDEXX Laboratories) for the detection of progressive infection is 71.8%, which is similar to the PPV of 71.2% identified in the present study. Hence, a similar number of discordant results would be expected, particularly in a group of cats with conditions commonly associated with FeLV infection. Nevertheless, the high discordancy rate identified here emphasizes the need for follow-up FeLV PCR testing.^18^

In the population discussed here, there is a difference in the validity of the test and the validity of the interpretation. Given the performance characteristics of the tests, it is unlikely that either the antigen or PCR tests were truly ‘incorrect’, but it is rather more likely that they were performing as intended, and it is the clinical interpretation that must be adjusted. Whether this population of cats had regressive or focal FeLV infection or they were negative with an alternative cause of discordancy is unclear; however, it is evident that some cats with clinical signs of hematologic disease and positive FeLV antigen testing do not have classic progressive infection, which must be considered in treatment decisions.

## Conclusions

This report describes the case characteristics of 21 cats with bone marrow disease or marked marrow activation and LFI/ELISA FeLV test results that were discordant with PCR results. Sixteen cats improved with therapy and seven tested negative on a subsequent ELISA. Therefore, it is considered likely that the initial LFI/ELISA result was not reflective of progressive FeLV infection, despite consistent clinical and hematologic signs. The cause of the discordant results is unknown; however, it appears that marked marrow disease can be associated with a positive FeLV LFI/ELISA result without evidence of progressive infection. These findings should be considered in cats with positive FeLV immunoassay results and marrow disease, and affirm the importance of follow-up PCR testing.
